# Proposed definition of competencies for surgical neuro-oncology training

**DOI:** 10.1007/s11060-021-03750-6

**Published:** 2021-04-21

**Authors:** Marcel A. Kamp, Bastian Malzkorn, Christiane von Sass, Francesco DiMeco, Constantinos G. Hadjipanayis, Christian Senft, Marion Rapp, Irina Gepfner-Tuma, Konstantinos Fountas, Sandro M. Krieg, Martin Neukirchen, Ioan Ștefan Florian, Oliver Schnell, Hendrik-Jan Mijderwijk, Alessandro Perin, Peter Baumgarten, Jasper H. van Lieshout, Niklas Thon, Miriam Renovanz, Ulf Kahlert, Jochem K. H. Spoor, Daniel Hänggi, Aaron Lawson McLean, Matthias Mäurer, Silvio Sarrubbo, Christian F. Freyschlag, Nils O. Schmidt, Francesco Vergani, Christine Jungk, Marco Stein, Marie-Therese Forster, Jeffrey S. Weinberg, John Sinclair, Evgenii Belykh, Lorenzo Bello, Emmanuel Mandonnet, Aliasgar Moiyadi, Michael Sabel

**Affiliations:** 1 Department of Neurosurgery, Centre of Neuro-Oncology, Jena University Hospital, Friedrich-Schiller-University Jena, Am Klinikum 1, 07747 Jena, Germany; 2 Centre of Neuro-Oncology, Department of Neurosurgery, Medical Faculty, Heinrich-Heine-University Düsseldorf, Moorenstraße 5, 40225 Düsseldorf, Germany; 3 Medical Education, Office of the Deanery of the Faculty of Medicine, Heinrich-Heine-University Düsseldorf, Düsseldorf, Germany; 4 Department of Pathophysiology and Transplantation, University of Milan, Milan, Italy; 5 Department of Neurological Surgery, Istituto Nazionale Neurologico “C. Besta”, Milan, Italy; 6 Department of Neurosurgery, Icahn School of Medicine at Mount Sinai, Mount Sinai Health System, New York, USA; 7 Brain Tumor Nanotechnology Laboratory, Tisch Cancer Institute, New York, USA; 8 Hans Berger Department of Neurology, Jena University Hospital, Jena, Germany; 9 Department of Neurosurgery, University Hospital of Larissa, Larissa, Thessaly Greece; 10 Medical School, University of Thessaly, Thessaly, Greece; 11 Department of Neurosurgery, Klinikum Rechts Der Isar, Technische Universität München, Munich, Germany; 12 Interdisciplinary Centre for Palliative Care, Medical Faculty, Heinrich-Heine-University Düsseldorf, Düsseldorf, Germany; 13 Department of Anesthesiology, Medical Faculty, Heinrich-Heine-University Düsseldorf, Düsseldorf, Germany; 14 Department of Neurosurgery, Cluj County Emergency Hospital, Cluj-Napoca, Romania; 15 Department of Neurosurgery, University of Medicine and Pharmacy “Iuliu Hatieganu”, Cluj-Napoca, Romania; 16 Department of Neurosurgery, Medical Centre, University of Freiburg, Freiburg, Germany; 17 Neurosurgical Clinic, University of Munich (LMU), Campus Grosshadern, Munich, Germany; 18 Department of Neurology & Neuro-Oncology, University Hospital Tuebingen, Tuebingen, Germany; 19 Department of Neurosurgery, University Hospital Tuebingen, Tuebingen, Germany; 20 Division of Preclinical Neuro-Oncology, Department of Neurosurgery, Medical Faculty, Heinrich-Heine-University Düsseldorf, Düsseldorf, Germany; 21 Departments of Neurosurgery, Erasmus MC, University Medical Center Rotterdam, Rotterdam, Netherlands; 22 Blizard Institute, Barts and the London School of Medicine and Dentistry, Queen Mary University of London, London, UK; 23 Department of Radiation Oncology, Jena University Hospital, Jena, Germany; 24 Department of Neurosurgery, Structural and Functional Connectivity Lab Project, Azienda Provinciale Per I Servizi Sanitari (APSS), Trento, Italy; 25 Department of Neurosurgery, Medical University of Innsbruck, Innsbruck, Austria; 26 Department of Neurosurgery, University Medical Centre Regensburg, Regensburg, Germany; 27 Department of Neurosurgery, King’s College Hospital NHS Foundation Trust, London, UK; 28 Department of Neurosurgery, University Hospital Heidelberg, Heidelberg, Germany; 29 Department of Neurosurgery, Justus-Liebig University Giessen, Giessen, Germany; 30 Department of Neurosurgery, Goethe University Hospital, Frankfurt am Main, Germany; 31 Department of Neurosurgery, The University of Texas MD Anderson Cancer Center, Houston, TX USA; 32 Department of Neurosurgery, The Ottawa Hospital Civic Campus, Ottawa, ON Canada; 33 Department of Neurosurgery, New Jersey Medical School, Rutgers, NJ USA; 34 Neurosurgical Oncology Unit, Department of Oncology and Hemato-Oncology, Università Degli Studi Di Milano, Milan, Italy; 35 Department of Neurosurgery, Lariboisière Hospital, Paris, France; 36 Neurosurgical Services, Department of Surgical Oncology, Tata Memorial Centre and Homi Bhabha National Institute, Mumbai, Maharashtra India

**Keywords:** Surgical neuro-oncology, Neuro-oncology, Education, Entrustable professional activities, EPAs, Competencies, Competence-based learning

## Abstract

**Objective:**

The aim of this work is to define competencies and entrustable professional activities (EPAs) to be imparted within the framework of surgical neuro-oncological residency and fellowship training as well as the education of medical students. Improved and specific training in surgical neuro-oncology promotes neuro-oncological expertise, quality of surgical neuro-oncological treatment and may also contribute to further development of neuro-oncological techniques and treatment protocols. Specific curricula for a surgical neuro-oncologic education have not yet been established.

**Methods:**

We used a consensus-building approach to propose skills, competencies and EPAs to be imparted within the framework of surgical neuro-oncological training. We developed competencies and EPAs suitable for training in surgical neuro-oncology.

**Result:**

In total, 70 competencies and 8 EPAs for training in surgical neuro-oncology were proposed. EPAs were defined for the management of the deteriorating patient, the management of patients with the diagnosis of a brain tumour, tumour-based resections, function-based surgical resections of brain tumours, the postoperative management of patients, the collaboration as a member of an interdisciplinary and/or -professional team and finally for the care of palliative and dying patients and their families.

**Conclusions and Relevance:**

The present work should subsequently initiate a discussion about the proposed competencies and EPAs and, together with the following discussion, contribute to the creation of new training concepts in surgical neuro-oncology.

**Supplementary Information:**

The online version contains supplementary material available at 10.1007/s11060-021-03750-6.

## Introduction

Improved and specific training in surgical neuro-oncology promotes neuro-oncological expertise, improves the quality of surgical neuro-oncological treatment, and may also contribute to further development of neuro-oncological techniques and treatment protocols. However, neither specific curricula for a surgical neuro-oncologic education nor a common consensus on the mandatory and optional content of neuro-oncological training have yet been established. Modern education theories emphasize acquisition of core competencies rather than the transfer of pure knowledge or skills alone. Therefore, it is compulsory to define the competencies needed prior to creating a corresponding curriculum.

Hence the aim of this work is to define the skills that should be acquired within the framework of surgical neuro-oncology training. The present work should initiate a discussion about this and, together with the following research, will contribute to the creation of new training methods and paradigms.

## Methods

We used a consensus-building approach, similarly to the one described by Vergouwen et al., 2010 [1]. Initially, the corresponding author (M.A.K.) developed the idea of defining specific competencies for education in neuro-oncology and discussed the idea during the 2019 “Neuro-Oncology” section meeting of the German Society of Neurosurgery. He proposed a draft with definitions of competencies and discussed it with a specialist in medical education of the medical faculty, Heinrich-Heine-University Düsseldorf. He identified and contacted a group of national and international experts in the field of surgical neuro-oncology that include Europe and North America. Based on the suggestions of this group, further authors were invited to contribute resulting in a group of 28 experts in the field. We implemented the suggestions of all co-authors and then sent the manuscript back to them for review. M.A.K. conveyed this process and repeated it four times until a consensus was reached between all authors.

### Considerations for the definitions of competences in surgical neuro-oncology

#### Definition of the term “competency” and “levels of skills”

“Medical competency” is a well-established term in the context of medical education, as opposed to medical expertise. The definition of medical competency was developed alongside the introduction of competency-based medical education. Since conveying purely theoretical knowledge is considered insufficient by modern medical standards, newer education methods aim to overcome this approach and move towards teaching, sharing and assessing competencies. Epstein and Hundert established a common definition of professional competencies [2] as encompassing “habitual and judicious use of communication, knowledge, technical skills, clinical reasoning, emotions, values, and reflection in daily practice for the benefit of the individual and community being served” [2]. Competency therefore comprises the integration of knowledge, skills, and attitudes required for successful and responsible problem-solving in various situations. Currently, these definitions together with competency-based medical education are the basis for several frameworks of medical education such as the Canadian CanMEDS framework [3, 4], the Scottish Deans’ Medical Curriculum [5], the Australian Curriculum Framework for Junior Doctors [6], the 2019 Framework for Undergraduate Medical Education in the Netherlands [7], the German National Competence Based Catalogues of Learning Objectives for Undergraduate Medical Education (NKLM) [8], and The Accreditation Council on Graduate Medical Education (ACGME) in the US [9].

Medical theorists have established various models describing different levels of skills. One of the most influential models is Miller’s pyramid. George Miller defined four hierarchical steps: knows (knowledge), knows how (competence), shows how (performance) and finally does (action) [10]. Based on this model and the Swiss Catalogue of Learning Objectives for Undergraduate Medical Training (SCLO), the NKLM defined the following levels of skills [8]:

- *Factual knowledge*: Descriptive knowledge of facts
- *Reasoning*: Explanation of facts, relationships, their classification in a clinical-scientific context and evaluation on a data driven basis.
- Perform under supervision.
- Act independently while being aware of potential consequences.

### Definition of the term “entrustable professional activities” (EPAs)

The concept of competencies focuses on individuals and their knowledge, skills, and attitudes. A practical problem when teaching skills is how to operationalize professional tasks and when to delegate professional activities. Additionally, medical education should target “standardised levels of proficiency to guarantee that all learners have a sufficient level of proficiency at the completion of training” [11, 12]. Entrustable professional activities (EPAs) is a model that defines requirements for trainees for the execution of practical activities. EPAs are defined as observable and measurable units of professional practice (key task) in a given (sub-) specialty [11, 13]. In contrast to competencies focusing on individuals and their abilities, EPAs focus on operationalising, acquiring and examining professional activities. They are not an alternative to competencies but rather incorporate and complete them [13]. EPAs comprise of different competencies. In North America in particular, milestones as a definition of different levels of a profession are common [14–17]. These levels serve as an observable marker of an individual’s ability [11].

As for competencies, different depths of proficiencies are defined for EPAs. A five-level entrustment scale is common. It defines the following levels [11, 18]:

1. Observation without allowance to practice EPAs
2. Execution of EPAs only under proactive, direct supervision
3. Execution of EPAs only under reactive/on-demand supervision
4. Execution to practice EPAs unsupervised
5. Supervision of trainees in practicing EPAs

### Various target groups and levels of skills for education in neuro-oncology

Education in surgical neuro-oncology targets different interest groups and therefore has different contents, objectives, and aims. Depending on the target groups, objectives and skills are taught in different depths.

As far as the education of medical students is concerned, it should only address those key-competencies in the field of neuro-oncology which are relevant for general medical practice. Knowing how to conduct a neurological examination, which alterations to expect in case of CNS lesions and a sound basic knowledge of neurology, neurosurgery, -pathology, -radiology and radiation oncology will give them an overview helping to diagnose their future patients and steer them in the right direction. In order to convey this, neurology and neuro-radiology have to become part of the core curriculum in medical school. To optimize time management, students who are committed to a neurosurgical path early on in their career could be given the opportunity—after having completed the required amount of time on the medical/neurological ward—to maximise their time on the neurosurgical service/theatre and attend targeted courses. Moreover, incorporating neuro-oncological topics into general medical education might give students an example for working in a multidisciplinary team and offers the opportunity to develop knowledge in dealing with colleagues from a number of disciplines. These skills will become more important as our collective knowledge increases, and the medical field continues to subdivide into sub-specializations [19]. The interdisciplinary nature of neuro-oncology might also be reflected by interdisciplinary neuro-oncological didactic sessions, e.g. joint seminars given by neurosurgeons, neurologists, radiation oncologists, neuroradiologists and/or neuropathologists. Depending on the educational framework of each medical school, the subspecialty of surgical neuro-oncology gives the opportunity to confront and discuss ethical medical problems with students. This can either be done in neuro-oncological lectures or seminars or in a more general framework, e.g. in ethical or palliative care courses or conferences. Examples for the integration of neuro-oncological teaching content into medical studies are interdisciplinary neuro-oncological lectures (e.g. inverted classroom format), interdisciplinary discussions of relevant topics (e.g. discussion of the subcortical fibre tract anatomy from a neuroanatomical and -surgical point of view), or interdisciplinary neuro-oncological elective seminars in addition to participating in interdisciplinary, ethical or palliative case-based discussions.

In general, medical students and young trainees should have a descriptive knowledge of relevant facts and explain facts and relationships in a clinical-scientific context on a data driven basis (skill level 1 and 2). The “Socratic” method may be a good way to hear their thoughts and may be implemented during case conferences [20].

When considering specialty training, specific neuro-oncological skills and surgical techniques become paramount. Residents and neurosurgeons who are not specialized in neuro-oncology need and must have a profound basic neuro-oncological knowledge and should be able to manage uncomplicated neuro-oncological patients and perform simple tumour operations independently. In contrast to resident teaching, educating a specialist (e.g. during a fellowship) sets a different goal: teaching the overall competency to independently treat patients with complex neuro-oncological diseases and being aware of potential consequences. Fellows and seniors are expected to firstly perform under supervision and later to act independently while being aware of potential consequences, respectively (skill level 3A and B).

As a result, education in neuro-oncology addresses different target groups with different skills and a different level of competence/performance.

### Proposed competencies to be achieved in the education of surgical neuro-oncology

We defined a total of 70 competencies in the fields “human basic factors”, “neuro-pathology, -anatomy, -physiology”, “diagnostics”, “surgical treatment”, “non-surgical treatment” and “others”. Table 1 summarizes the proposed competencies to be achieved.

**Table 1:** Domains and their core content of the defined competencies

*Human basic factors*
Prioritization of optimal patient care and team needs over personal need
Recognition of own limitations and seeking help from other team members
Adequate, appropriate, clear and concise communication even in emotionally challenging situations including delivering bad news appropriately
Advising patients and their relatives on neuro-oncological diseases
Work and cooperate constructively in a (multi-professional) team
*Neuropathology, -anatomy, -physiology*
Knowledge of the neuropathology of brain tumours and their classification
Expertise of the topographical and functional neuroanatomy, in particular the cortical and subcortical localization of neurofunctions
Knowledge of the neurophysiology of functional neuronal systems (e.g. language function)
Expertise on the arterial and venous anatomy of the central nervous system
*Diagnostics*
Detection and management of neuro-oncological emergencies
Prioritization of urgent neuro-oncological/medical issues
Able to perform a systematic neurological examination and consequently assign deficits to lesions and neuroanatomical and -physiological concepts appropriately
Initiation and assessment of diagnostic and radiologic procedures and knowledge of their possibilities, limitations and risks
Independent indication and execution of invasive diagnostic procedures (lumbar punctures, biopsies)
*Surgical treatment*
Indication of different operative therapies and methods
Selection and planning a suitable surgical procedure
Respect of and adherence to established procedures and local safety protocols
Safe and skilled application of different surgical techniques in neuro-oncology with a timely performance (e.g. approaches, microsurgical techniques, neuro-navigation)
Application of surgical techniques for intraoperative assessment of the degree of surgical resection (e.g. fluorescence, iMRT, ultrasound) and techniques for intraoperative localisation of neuronal function (intraoperative neurophysiological monitoring, awake surgery)
Mastering, properly discussing, and reporting complications
*Non-surgical treatment*
Monitoring, time-sensitive interventions and management in critically ill patients
Competency in postoperative/intensive care of neuro-oncologic patients
Indications and contraindications of standard neuro-oncological adjuvant therapies
Detailed knowledge about/indication of adjuvant oncological therapies
Indications/advice on basic features of other, alternative non-operative therapy procedures
Knowledge about basic features of radiation oncology including knowledge about/skills in stereotactic radiosurgery, proton therapy, intraoperative radiation therapy
Initiation and basic knowledge on psycho-oncological therapy including screening methods
Timely initiation of palliative care (e.g. early integration) and basic knowledge about standard concepts in palliative care
*Other competencies*
Detailed knowledge of common guidelines, recommendations and relevant literature
Usage of scientific and other evidence-based resources
Appreciating the importance of both basic and clinical research; assess, apply and translate new knowledge and practices
Knowledge, respect of and adherence to established ethical standards and laws
Basic competence in both basic and clinical research applied to neuro-oncology

The table gives an overview of the domains and their core content of the defined competencies. A complete list of the defined competencies is provided in the supplement

**Table 2:** Overview about the defined EPAs with required key knowledge, skills and attitudes required

Knowledge	Skills	Attitude
*EPA 1: Non-operative management of patients with a brain tumour diagnosis*		
Knowledge about the neuropathology of brain tumours	Adequate communication and collaboration	
Diagnostic work-up and interdisciplinary treatment	Advising patients and their families	Considering the patient´s wishes
Consideration of common differential diagnoses	Management of critically ill patients	Prioritization of urgent medical issues
	Coordination of interdisciplinary and interprofessional assessments	Recognition of own limitations
*EPA 2: tumour-based resection*		
Expertise of anatomy of the brain	Adequate and appropriate communication/cooperation	
Knowledge about the neuropathology/techniques for tumour removal	Correct indication of surgery balancing risks and benefits	Recognition of own limitations and willingness to seek for help
Awareness of different surgical goals	Mastering all required surgical techniques in a safe and efficient manner	Prioritization of urgent medical issues
Expertise in surgical techniques	Use of techniques for intraoperative localisation of tumour boundaries	Adherence to established institutional safety protocols
Awareness of different anaesthetic techniques		
*EPA 3: function-based surgical resection of brain tumours*		
Expertise of anatomy of the brain	Adequate and appropriate communication/cooperation	
Knowledge about the neuropathology and techniques for tumour removal	Adequate selection and planning of preoperative investigations risks and benefits	Create a constructive a motivating relationship with the patients
Knowledge about intraoperative monitoring and intraoperative neurological testing	Choosing a suitable surgical procedures fe and efficient manner	Respect for and adherence to established procedures and institutional safety protocols
Expertise in surgical techniques	Apply and correctly interpret intraoperative monitoring	
Awareness of different anaesthetic techniques	Indicate, prepare and master awake surgeries	
Balance the targeted extent of resection to the functional risks	Effective management of limitations and complications	
*EPA 4: postoperative management of brain tumour patients*		
Knowledge about the neuropathology molecular markers	Adequate and appropriate communication/cooperation	
Knowledge about signs, symptoms and management of complications	Systematic approach to patient assessment and therapy	Create a constructive a motivating relationship with the patients
Diagnostic work-up and inter-disciplinary treatment of patients	Evaluation of post-operative imaging	Respect for and adherence to established procedures and institutional safety protocols
Knowledge about multimodal treatment plans and radiation oncology treatment methods	Coordination of interdisciplinary treatment plans	
Consideration of common differential diagnoses of postoperative neurological deterioration	Delivering bad news appropriately (resource- activating, supportive)	
Postoperative imaging of brain tumour patients	Advising patients and their families	
*EPA 5: management of deteriorating brain tumour patients*		
Recognise red flags and emergencies	Clear, concise and structured communication	
Consider common differential diagnoses	Systematic approach to patient assessment and therapy	Usage of an appropriate level of urgency for further management
Initiate time-critical further monitoring, assessment and therapy	Skills in emergency medicine and neuro-oncological surgery	Recognition of own limitations and when to seek for help
	Adequate judgement on the need of an implementation of further therapy	Prioritization of urgent medical issues
	Clear, concise and structured communication	Calm demeanour
*EPA 6: early palliative care for dying patients and their families*		
Treatment of symptoms and suffering on four symptom levels	Adequate and appropriate communication/cooperation	
Knowledge about palliative anti-tumour therapies	Advising patients and their relatives	Cooperation in a (multi-professional) team and constructive teamwork
Recognize physical signs and symptoms of dying patients	Delivering bad news appropriately, taking a conversation model into account	Appreciating the importance and time sensitivity in treating palliative humans
Criteria for when to start palliative care (e.g. early integration)	Coordinating interdisciplinary and interprofessional assessments	Establish ethical principles and apply them to end-of-life care
knowledge about palliative care structures	Recognizing dying patients and treating them within standardized procedures	
*EPA 7: collaboration as a member of an interdisciplinary and/or -professional neuro-oncology team*		
Factors that affect teamwork and effective communication	Actively strives to integrate into the team	Feels committed to the goal of the team and optimal patient care
Strategies for safe communication	Adequate and appropriate communication	Prioritizes an optimal patient care and team needs over personal needs
	Establishes a climate of respect, appreciation, integrity, and trust	Recognizes the role, responsibilities, contributions and value of all team members
		Offers help to members of the team in need
		Includes and attentively listens to team members and considers feedback
*EPA 8: basic and clinical research activity in neuro-oncology*		
Understanding of the main scientific challenges in Neuro-Oncology	Perform a literature review based on scientific libraries	Establish ethical principles and apply them in research
knowledge about basic research methods and approaches	Translate problems into precise scientific questions	Knowledge, respect of and adherence to established ethical protocols
knowledge about statistical analysis and interpretation of data	Compile, analyse und interpret clinical and experimental data sets	Respect of and adherence to national and international law
	Preparing/etting up a clinical trial	
	Prepare scientific results for a specialist audience	

Table 2 gives an overview about the defined EPAs with key knowledge, skills and attitudes for each EPA. A detailed definition of each EPAs including a specification, a definition of all required knowledge, skills and attitudes and recommended potential assessment tools to evaluate progress and proficiency are provided in supplement

*IOM* intraoperative neurophysiological monitoring

### Proposed selection and definitions of entrustable professional activities for education and training in surgical Neuro-Oncology

We selected eight typical professional key tasks in surgical neuro-oncology. Based on these typical tasks and the competencies mentioned above (see Table 1), eight EPAs were defined:

EPA 1: Non-operative management of patients with the diagnosis of a brain tumour

EPA 2: Tumour-based resection

EPA 3: Function-based surgical resection of brain tumours

EPA 4: Postoperative management of brain tumour patients

EPA 5: Management of deteriorating brain tumour patients

EPA 6: Collaboration as a member of an interdisciplinary and / or -professional neuro-oncology team

EPA 7: Early palliative care for dying patients and their families

EPA 8: Basic and clinical research activity

**Fig. 1:**
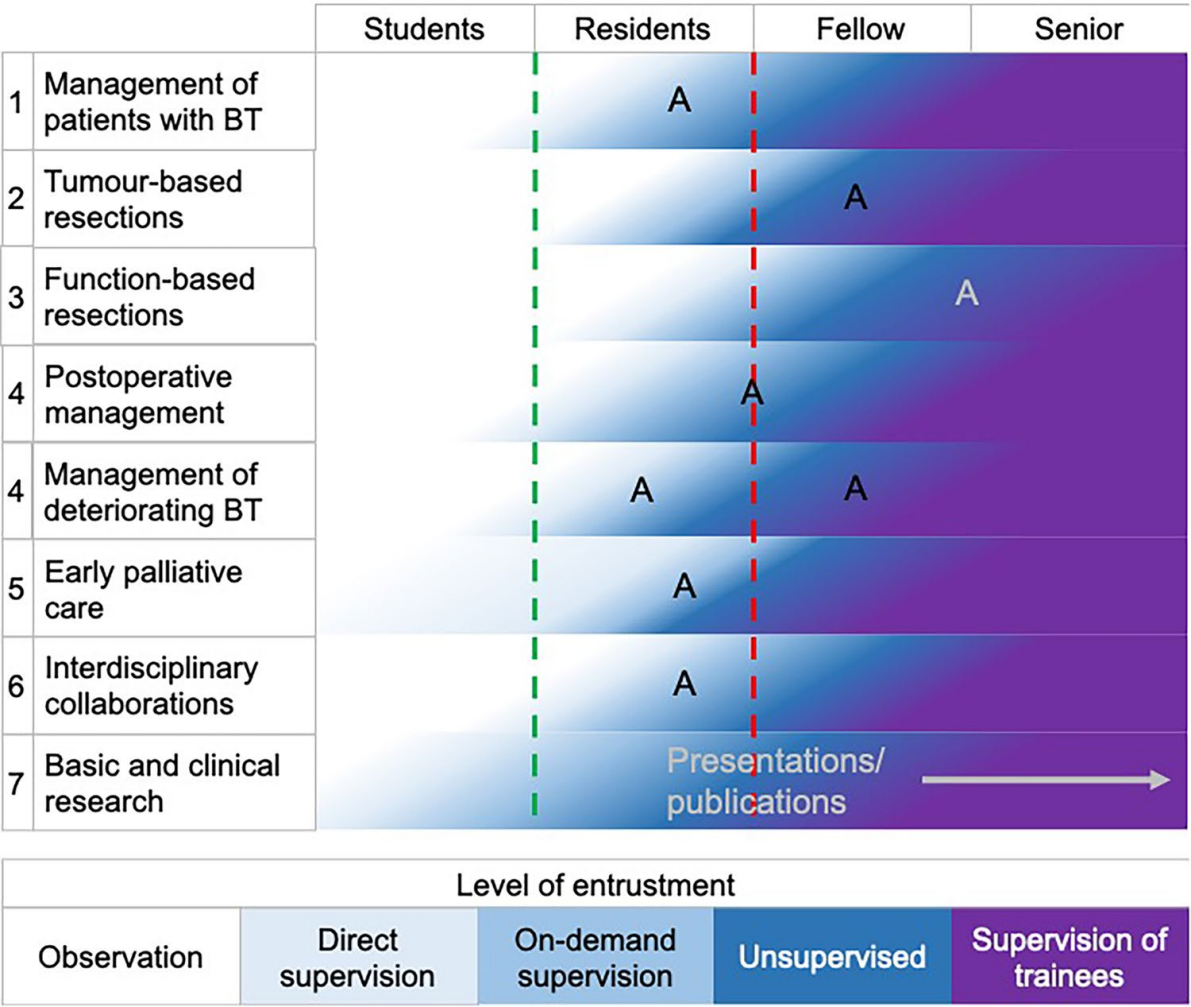
Levels of entrustment. Figure 1 visualizes degree of entrustment that should be achieved at various points in time during the training. The color-coding of the five-level entrustment scale is given at the bottom of the figure. The green dotted line indicates the licence to practise and the red dotted line the neurosurgical board examination. “A” indicates possible time-points for an assessment of proficiency in each EPA. However, time-point and form of the assessment should be adapted to the local frameworks and other qualifications as e.g. a neurosurgical board examination may be taken into account. *A* assessment, *BT* brain tumour

A detailed description of each EPA including information regarding relevant domains of competence and required knowledge, skills and attitudes are presented in the supplement (Supplement files 1 and 2). Evaluation and documentation of progress and proficiency during residency and fellowship are crucial. A detailed overview on recommended potential assessment tools is given in the supplement section. In general, progress should be discussed and documented during annual or 6-monthly meetings with the training director or supervisor. Documentation of progress can be facilitated by using an app (e.g. ACGME app in the U.S.). A wide range of assessment tools to document progress and proficiency can be used, depending on the EPA: case and procedure logbooks, direct observation of procedural skills (DOPS), anticipatory guidance, different forms of feedback or presentation and case-based discussions of patients and their management. Moreover, passing the exam of national board of neurological surgery (ABNS, EBNS or similar), attending courses or conferences and completing rotations in e.g. neuro-ICU, -anaesthesia, -pathology, clinical neurophysiology and neuro-oncological clinics documents experience and proficiency in specific areas, respectively. Expected progression of entrustment over the training period is summarized in Fig. 1.

## Discussion

The present consensus paper provides a detailed definition of competencies which we believe are essential for surgical neuro-oncology. EPAs for training in surgical neuro-oncology might contribute to the creation of new training concepts in surgical neuro-oncology.

What is expertise in surgical neuro-oncology? Traditionally, expertise is assumed once certain indicators have been met: years of experience, specialization including specialty board certification, to successfully complete a fellowship and courses, and/or academic rank or responsibility [21, 22]. Dreyfus and Dreyfus developed a model to define the different stages of expertise and skills in clinical medicine based on the education of pilots. According to this model there are five stages of knowledge and skills: novice, advanced beginner, competent, proficient and expert [23]. Experts are characterized by their clinical intuition, pattern recognition, and ability to adapt and react to disruptions in expected patterns [24]. Moreover, expertise in surgery is often related to the number of index procedures performed by a surgeon. In contrast, for surgical trainees, it is well known that the number of index procedures does not necessarily reflect their expertise [25]. Thus, more reliable tools for certification and assessment of expertise are required.

Speciality training in surgical neuro-oncology is mandatory to acquire specific knowledge and skills to diagnose and treat patients suffering from tumours involving the central nervous system and / or cranial or peripheral nerves. There are particular challenges specifically related to neuro-oncological education, such as the highly interdisciplinary nature of this field, which requires advanced knowledge in neuroanatomy, neuroradiology, neurophysiology, neuropathology, neurology, neurosurgery, nuclear medicine, medical as well as radiation oncology besides expertise in other areas, such as psycho-oncology and palliative care. Neuro-oncological topics and in particular aspects of surgical neuro-oncology might not be incorporated in the frameworks of many medical schools. Again, a prioritization on learning neurological examination and on learning the basics in neurology, neurosurgery, -pathology and -radiology might be much more relevant. However, some medical schools also address neuro-oncology as part of neurosurgical or neurological education while neuro-oncological training often only starts during the residency training for neurologists or neurosurgeons at other institutions. In addition, a small number of neurosurgical departments around the world offer special neuro-oncology or surgical neuro-oncology fellowships for specialist surgeons or doctors, while some neuro-oncological courses and training events (such as the interdisciplinary courses of the European Association of Neuro-Oncology or the Society of Neuro-Oncology) are available worldwide. An important example for a specialist education in surgical neuro-oncology is the accredited fellowship program by the Committee on Advanced Subspecialty Training (CAST) program and the Society of Neurological Surgeons (SNS) offering neurosurgical oncology fellowships in many prestigious neuro-oncology centres in the U.S. Additionally, CAST newly accredited skull base fellowships will likely be available in 2022.

Residency and fellowship programs, e.g. the U.S. training programs, often prepare for the EPAs addressing management of brain tumour patients including deteriorating patients and tumour- and function-based resections. We additionally defined the EPA “Collaboration as a member of an interdisciplinary and/or -professional neuro-oncology team” as interdisciplinary and -professional collaborations are paramount for an optimal neuro-oncology treatment. Residents/Fellows should attend tumour boards, present patients and coordinate interdisciplinary and interprofessional assessments and draw up treatment plans. However, a climate of respect, appreciation, integrity, and trust is essential at every point in clinical cooperation, e.g. in the operating room or on the ward. EPA 6 addresses an early palliative care for dying patients and their families, also in parallel to standard anti-tumours therapies. Assessment, recognition and treatment of symptoms as well as the patient’s complex suffering (total pain concept) should be an integral part of oncological care. Finally, EPA 8 addresses basic and clinical research activities in neuro-oncology. Knowledge about clinical research methods, statistical analysis and interpretation of data is necessary to interpret basic and clinical neuro-oncological research and finally to draw up up-to-date treatment plans. Moreover, some important and potentially ground-breaking treatments are only possible within clinical studies. Research electives might be one way to achieve competence in basic and clinical research.

We hope that the present definition may help to develop new competence-based teaching concepts for education in surgical neuro-oncology. Defining competencies and EPAs to be taught as well as the subsequent aims of neuro-oncologic education is crucial for an outcome-based education. All learning activities should be geared towards the intended learning outcome. According to the model of constructive alignment by John Biggs [26], all components in the teaching system have to be aligned, including the framework and its desired outcome as well as teaching and assessment methods. Choosing the appropriate objective assessment method of EPAs and giving constructive and relevant feedback (and hence aligning education of practical competencies and skills) may prove to be especially challenging. Many education frameworks are based on traditional teaching methods while few countries have EPAs and/or education in surgical neuro-oncology integrated in their neurosurgical training framework (e.g. the Netherlands, Canada and the United States). The Royal College of Surgeons of Canada established a competency-based design curriculum for all trainees entering training since 2019. This in itself has an outline of EPAs pertaining to neuro oncology although considerably general in nature. Certainly, the EPAs do not delve into such specific concepts as awake surgery, subcortical direct electrical stimulation or function-based resection which are at the forefront of current neuro-oncological surgery. The US Neurological Surgery Milestone Project established by a joint initiative of the Accreditation Council for Graduate Medical Education and the American Board of Neurological Surgery defines different milestone descriptions essential for neurosurgical education. This program also includes one milestone description for “Brain Tumor—Patient Care” with a definition of 3–5 items for the five entrustment levels. Moreover, EPAs and competencies defined in the present work and the definitions of expertise in neurosurgical oncology by the CAST program might have some overlap. These overlaps provide support for the new set of EPSs and we do not understand our proposal as competition but as a complement to the existing training concepts. Our training concept provides, on the one hand, a more detailed description of competencies essential for brain tumour treatment on a regular neurosurgical basis and, on the other hand, a framework of how a sub-specialization in surgical neuro-oncology can be achieved beyond neurosurgical training concepts.

The competencies and EPAs proposed here should not be deemed as exhaustive or infallible, but rather as a source of ideas and a basis for further discussion. Therefore, the defined competencies and EPAs certainly do not represent the entire scope of surgical neuro-oncology. Surgical neuro-oncology training seems to place emphasis on intrinsic brain tumours and cerebral metastases at most places. However, surgical treatment of spine, skull base and peripheral nerve tumours is an integral part of operative neuro-oncology. Adequate management and training of these lesions is mandatory and may later be integrated into a neuro-oncological framework. For various reasons we have deliberately refrained from further definitions: Treatment of intrinsic brain tumours and other tumours are provided by different teams in several institutions and the definition of training standards would have to involve further specialist societies (e.g. in Germany the German Society for Spine Surgery for spine tumours or the Society of Skull Base Surgery for skull base tumours). We aimed to limit the number of EPAs in the present work in order to possibly increase the acceptance of the project. For this reason, we have focused on the intrinsic brain tumours in the present work and recommend adding further EPAs at a later date.

Neuro-oncological practice and education across the world varies significantly among different countries and neurosurgical departments and might depend on socio-economic conditions as well as the availability of appropriate infrastructure commensurate with the disease burden. Not all equipment and techniques described might be available in every centre treating neuro-oncological patients. Rather, the tools and methods used depend on local treatment concepts, which may differ gradually. We also acknowledge that cultural differences regarding treatment decisions do exist between countries. However, the authors consider it essential that local concepts, tools and techniques enable the treatment of neuro-oncologic patients based on the EPAs and competencies described. In particular, tumour-based and function-based surgical resection of brain tumours should be possible in a safe, timely, skilful, and efficient manner in highly specialised neuro-oncological units. Moreover, skills like communication and empathy are equally important.

The aim of this work was to draw attention to the importance of training concepts in neuro-oncological teaching and of course contribute to improvements in surgical neuro-oncological training. To this end, from our point of view, we have defined important teaching contents in the form of competencies and EPAs, determined a time frame by when these competencies should be achieved and and made suggestions for potential assessment tools to evaluate progress and proficiency. However, we included little recommendations about how to teach the competencies. Again, the way of integration of competencies and EPAs in frameworks must be adapted to local conditions. Of course, we are also aware that implementing and operationalizing our recommendations is certainly a Herculean task. In addition, we left it open as to what should be done if someone does not achieve competencies. In general, a senior expert in neuro-oncology should act independently while being aware of potential consequences in all defined fields of competencies. In contrast, residents and neurosurgeons who do not specialize in neuro-oncology should have some basic competency, but do not have to master all competencies and EPAs independently (e.g. Function-based surgical resection of brain tumours).

Finally, an improved and more structured education in surgical neuro-oncology might attract more young colleagues, boost students’ and trainees’ satisfaction, advance expertise in surgical neuro-oncology, establish fellowships, and subsequently enhance patient care as well as promote surgical neuro-oncological techniques and concepts. Moreover, this teaching approach offers the possibility to impart aspects, skills, and attitudes that do not arise through conventional teaching approaches. Examples include reflected self-assessments, specific operational skills, teamwork, and constructive as well as appreciative communication with colleagues. Finally, a standardized EPA-based training in surgical neuro-oncology facilitates international collaboration, exchange and, again establishment of specific fellowships.

## Supplementary Information

Below is the link to the electronic supplementary material.

Supplementary file1 (XLSX 42 kb)

Supplementary file2 (DOCX 53 kb)

## Data Availability

The manuscript has no associated data.
