# Supplementary material for: Proposed definition of competencies for surgical neuro-oncology training
Source: J Neurooncol. 2021 Apr 21;153(1):121–31. doi: 10.1007/s11060-021-03750-6 (PMC8131302; doi:10.1007/s11060-021-03750-6)
Supplement: Supplementary file 2 — Supplementary file2 (DOCX 53 kb) [file 11060_2021_3750_MOESM2_ESM.docx]

**EPA 1: Non-operative management of patients with a brain tumour diagnosis**

**1. Title of the EPA**

*Non-operative management of patients with a brain tumour diagnosis*

**2. Specification and limitations**

The aim of this EPA is to reliably diagnose patients suffering from a brain tumour. Trainees should be able to consider and – if possible – exclude common differential diagnoses and develop an evidence-based individual treatment plan for the patient concerned. This plan should be based on the recommendations of common up-to-date guidelines and detailed knowledge about diagnostic and therapeutic options, their limitations and risks. It should be discussed with an interdisciplinary team that should take into account the informed patient's requests and wishes.

**3. Most relevant domains of competence**

The concept of EPAs is used as a framework that aims to teach very specific competencies and professional activities rather than knowledge alone. Pre-defined professional activities should be taught and undertaken under direct supervision only until a standardised level of proficiency has been reached and both teacher and trainee are confident with the performance.

This EPA represents a key task of neuro-oncological practice. Competencies defined in table 1 apply to this EPA.

**4. Required knowledge, skills and attitude**

*Knowledge:*

- Detailed knowledge about the neuropathology of brain tumours including the impact of relevant molecular markers and the patient´s clinical presentation and general condition
- Knowledge of the natural history such that a trainee understands the short survival without treatment (i.e. natural history) in order to effectively arrange diagnostic tests, inter-disciplinary consults and treatment in order to minimize the risk of further neurological decline/death.
- Necessary diagnostic work-up and interdisciplinary treatment of patients having been diagnosed with a brain tumour
- Consideration of common differential diagnoses and how to exclude them.

*Skills*:

- Systematic approach to patient assessment and therapy
- Management of critically ill patients, if necessary
- Coordination of interdisciplinary and interprofessional assessments, treatment and follow-up plans
- Adequate communication skills to initiate further assessments, consultation and further interdisciplinary therapy
- Adequate collaboration in a multidisciplinary and -professional team
- Appropriate delivery of the diagnosis in a resource-activating and supportive manner
- Advising patients and their families on neuro-oncologic diseases, their symptom load, prognosis, and operative and non-operative therapies based on up-to-date studies
- Considering the patient´s wishes

*Attitude:*

- Adequate and appropriate communication, cooperation in a (multi-professional) team and constructive teamwork
- Recognition of own limitations and when to seek for help from other team members to guarantee an optimal patient care
- Prioritization of urgent neuro-oncological/medical issues

**5. Recommended potential assessment tools to evaluate progress and proficiency as a standardised, summative entrustment decision**

Tools used for assessment and documentation of progress as well as for a standardised, summative entrustment decision should be adapted to local structures and educational frameworks. However, according Bigg´s model (1996) of constructive alignment, assessment methods should align with the desired outcome (teaching of practical activities) and the teaching format used (EPAs). In our view, the following assessment tools might be suitable for this EPA:

- Standardised feedback regarding management decisions from a neuro-oncological consultant and other medical professionals (e.g. consultant of other medical disciplines, nurses)
- Direct Observation of Procedural Skills (DOPS)
- Supervisor feedback
- 360° feedback
- Peer assessment
- Chart Review
- Procedure Logbook
- Presentation and discussion of patients and their management in person, or over the phone
- Anticipatory guidance – ‘what if’ discussion with a consultant to explore ability to cope with other scenarios.
- Attending corresponding courses (e.g. corresponding courses of the German Society of Neurosurgery)
- Knowledge may be tested by passing the U.S written boards or equivalent exam
- Learning outcome-based rotations in neuro-ICU, anaesthesia, neuro-pathology, clinical neurophysiology and neuro-oncological clinics

**6. Entrustment for level of supervision necessary at each stage of training**

The entrustment of the professional activity should be adapted to local structures and educational frameworks.

**EPA 2: Tumour-based resection**

**1. Title of the EPA**

*Tumour-based resection*

**2. Specification and limitations**

The aim is to implement an EPA to master the independent tumour-based surgical management. This EPA focuses on standard techniques and tools of (micro)-neurosurgery and on the adequate use of tools aiming to identify tumour-boundaries preoperatively (MRI sequences, PET, Spectroscopy) and intraoperatively (neuronavigation, ultrasound, 5-ALA, iMRI or iCT, ...). This EPA is aimed at neurosurgical trainees/resident physicians, in particular those with a special interest in surgical neuro-oncology. Step by step, knowledge, skills and attitude for a safe and efficient conduct of neuro-oncological surgeries have to be learnt. This includes – among other aspects – to correctly indicate operative therapies including obtaining an informed consent, planning of the surgery and deciding on an adequate selection of pre-operative diagnostics, of required intra-operative methods and tools, and the positioning of the patient. In addition, a safe, skilful and timely management of the surgical approach, surgical and micro-surgical techniques as well as applying intraoperative tools (e.g. for tumour localisation) will be required for this EPA. Of utmost importance is the knowledge of ones’ own limits, an early recognition of intraoperative problems and seeking for help of more experienced colleagues if necessary. Additionally, the trainee should be able to communicate adequately and effectively, and behave professionally. Institutional/local safety protocols and procedures should be known, respected and adhered to.

**3. Most relevant domains of competence**

The concept of EPAs is a framework that aims to teach very specific competencies and professional activities rather than knowledge alone. Predefined professional activities should be taught and undertaken under direct supervision only until a standardized level of proficiency has been reached and both teacher and trainee are confident with the performance.

This EPA represents a key task of neuro-oncological practice. Competencies defined in table 1 apply to this EPA.

**4. Required knowledge, skills and attitude**

*Knowledge:*

- Extensive knowledge about the topographical, structural, functional, and vascular anatomy of the brain, particularly the anatomy relevant to the surgical site
- Detailed knowledge about the neuropathological behaviour of different brain tumours and the specific techniques used for tumour removal.
- Knowledge of natural history be again emphasized in order to effectively arrange surgery and adjuvant treatment(s) in a ‘timely fashion’ in order to minimize the risk of further neurological decline/death.
- Awareness of different surgical goals (resection, open biopsy, stereotactic biopsy) and ability to weigh pros and cons in the individual patient’s situation.
- Knowledge about the impact of (maximized) resection for the clinical course in the context of suspected diagnosis (e.g. lymphoma vs. glioblastoma) at first diagnosis and during the course of disease.
- Expertise in preoperative identification of tumour-boundaries, patient positioning, surgical approaches, surgical and microsurgical techniques, techniques for intraoperative navigation, tumour identification and localisation, particularly techniques for intraoperative localisation of tumour boundaries (neuronavigation, ultrasound, 5-ALA, fluorescein, iMRI, iCT, ...) is needed.
- Understanding the use of conventional microscope and/or exoscope for brain tumour surgery
- For all techniques, limitations and complications should be known.
- Awareness of different anaesthetic techniques and their (potential) impact on surgery
- Ability to communicate effectively with the anaesthetist in charge
- Knowledge about the indication and suitable location for frozen section and tissue sampling

*Skills:*

- Independent and correct indication of surgery balancing risks and benefits
- Selection and planning of necessary preoperative investigations, suitable surgical procedures / methods, positioning, and surgical approaches
- Mastering all required surgical techniques in a safe, timely, skilful and efficient manner - potential complications should be identified beforehand and skillset to control arising complications acquired.
- In particular adequate use of techniques for intraoperative localisation of tumour boundaries (neuronavigation, ultrasound, 5-ALA, Fluorescein, iMRI, iCT, ...)
- Safe and skilled application of different (micro-) surgical techniques should be trained before operating on patients. This could e.g. be done on models (sheep brain tumour model), on 3D haptic feedback simulators, and/or on surgical planners.

*Attitude:*

- Recognition of own limitations and willingness to seek for help from other team members to guarantee an optimal patient care
- Adequate and appropriate communication, cooperation in a (multi-professional) team and constructive teamwork, in particular a highly-effective and respectful cooperation in a multi-disciplinary and -professional team in the operating room
- Prioritization of urgent neuro-oncological/medical issues
- Respect for and adherence to established procedures and institutional safety protocols
- Harvesting tumour tissue for biobanking and enrolment into clinical trials

**5. Recommended potential assessment tools to evaluate progress and proficiency as a standardised, summative entrustment decision**

Tools used for assessment and documentation of progress as well as for a standardised, summative entrustment decision should be adapted to local structures and educational frameworks. However, according Bigg´s model (1996) of constructive alignment, assessments methods should align with the desired outcome (teaching of practical activities) and the teaching format used (EPAs).

Before operating on neuro-oncological patients, safe and skilled application of different (micro-) surgical techniques should be trained on models (sheep brain tumour model), on 3D haptic feedback simulators, and/or on surgical planners. These skills and specific knowledge as defined above should be examined before operating on patients.

The following tools might be suitable for an such an assessment:

- Direct observation of procedural skills (DOPS)
- Standardised feedback regarding management decisions from a neuro-oncological consultant and other medical professionals (e.g. consultant from other medical disciplines, nurses).
- Supervisor feedback
- 360° feedback
- Peer assessment
- Chart review
- Procedure logbook
- Presentation and discussion of patients and their management in person, or over the phone.
- Anticipatory guidance: “what if” discussion with a consultant to explore ability to cope with other scenarios
- Attending appropriate courses (e.g., of the German Society of Neurosurgery)
- Knowledge may be tested by passing the U.S written boards or equivalent test
- Learning outcome-based rotations in neuro-ICU, anaesthesia, neuro-pathology, clinical neurophysiology and neuro-oncological clinics

In our view, the following assessment tools might be suitable for an intraoperative assessment for this EPA:

- DOPS
- Attending appropriate courses (e.g., of the German Society of Neurosurgery)
- CbD (Case based Discussions)

**6. Entrustment for level of supervision necessary at each stage of training**

The entrustment of the professional activity should be adapted to local structures and educational frameworks.

**EPA 3: Function-based surgical resection of brain tumours**

**1. Title of the EPA**

*Function-based surgical resection of brain tumours*

**2. Specification and limitations**

The aim is to implement an EPA to master the independent surgical management of eloquent tumours. The basic surgical techniques of a tumour-based surgery have been outlined in the EPA “tumour-based resection”. In contrast to EPA 3, this EPA focuses on the technique of function-based surgical resection of brain tumours in both the asleep and awake craniotomy patient. This EPA aims to

- pre- and intra-operatively identify and preserve functional cortical areas and critical subcortical pathways surrounding brain tumours,
- learn about and safely apply techniques for pre-operative (tractography, task-based fMRI, TMS, MEG, resting state fMRI, …) and intra-operative (evoked potentials, direct electrical stimulation for cortical/subcortical mapping of motor pathways and cognitive functions in the anaesthetized and the awake patient) identification of cortical and subcortical critical functional nodes,
- understand and apply principles of pre- and post operative neuropsychological assessments and in case of awake surgery selecting the corresponding intra-operative task for neuro-cognitive monitoring.

These techniques enable to operate on infiltrating brain tumours and the surrounding infiltrated but potentially functional brain tissue. Function-based surgical resections of brain tumours pose a special challenge. In order to prevent the patients from peri-operative neurological deficits, it is mandatory to command a detailed knowledge of the functional cortical, subcortical, and vascular anatomy as well as an expertise in exact pre- and intraoperative localisation and thus preservation of neurological function.

**3. Most relevant domains of competence**

The concept of EPAs is a framework that aims to teach very specific competencies and professional activities rather than knowledge alone. Predefined professional activities should be taught and undertaken under direct supervision only until a standardized level of proficiency has been reached and both teacher and trainee are confident with the performance.

This EPA represents a key task of neuro-oncological practice. Competencies defined in table 1 apply to this EPA.

**4. Required knowledge, skills and attitude**

Knowledge, skills and attitudes described in the EPA “tumour-based resection” fully apply to this EPA. Additionally, the following knowledge, skills and attitudes are important for this EPA:

*Knowledge:*

- Extensive expertise in vascular, structural and functional cortical and subcortical anatomy
- Principles of neuropsychological assessment and selection of intra-operative tests for awake surgery with neuro-cognitive monitoring
- Knowledge about neurophysiological assessment techniques and the interpretation of pre- and intraoperatively acquired results as well as on their changes during surgery
- Basic knowledge about anaesthetic techniques and their impact on surgery and methods of functional testing (e.g. the effect of anaesthetics on intraoperative neurophysiological monitoring)
- Ability to balance the targeted extent of resection with the functional risks in the context of the suspected diagnosis and anticipated treatment plan.

*Skills:*

- Adequate selection and use of techniques for pre-operative identification of structural (tractography with updated protocols of acquisition and post-processing) and functional anatomy (task-based fMRI, resting-state fMRI, TMS, MEG).
- Adequate selection and planning of preoperative investigations
- Choosing a suitable surgical procedure/method to intraoperatively detect, test and preserve neuronal functioning
- Understand, apply and correctly interpret intraoperative neurophysiological monitoring in both the awake and asleep craniotomy patient
- Indicate, prepare and master awake surgeries, selection and application of intraoperative testing; Using these techniques for an intraoperative identification of functional boundaries
- Understand and correctly interpret intra-operative neuro-cognitive monitoring during awake surgery
- Effective identification and management of complications (e.g. intraoperative brain swelling, seizures).
- Manage limitations (e.g. neurological testing is not possible in awake) and – if necessary - draw the right conclusions (e.g. stop resection if an adequate identification of functional boundaries is not possible).

*Attitude:*

- Teams supporting an awake surgery tend to be larger, so adequate and efficient communication is paramount, as well as being part of a multi-professional team in the OR. The distribution of roles should be clear in this setting, teamwork constructive, respectful and efficient.
- Neurosurgeons involved in awake surgery should be able to create a constructive a motivating relationship with the patients involved.
- Urgent neuro-oncological or medical issues should be addressed clearly and managed appropriately.
- Established procedures and local/institutional safety protocols should be known, respected and adhered to.

**5. Recommended potential assessment tools to evaluate progress and proficiency as a standardised, summative entrustment decision**

Tools used for assessment and documentation of progress as well as for a standardised, summative entrustment decision should be adapted to local structures and educational frameworks. However, according Bigg´s model (1996) of constructive alignment, assessments methods should align with the desired outcome (teaching of practical activities) and the teaching format used (EPAs). In our view, the following assessment tools might be suitable for this EPA:

- Direct Observation of Procedural Skills (DOPS)
- Attending corresponding courses (e.g. corresponding courses of the German Society of Neurosurgery)
- Knowledge may be tested by passing the U.S written boards or equivalent exam
- Learning outcome-based rotations in neuro-ICU, anaesthesia, neuro-pathology, clinical neurophysiology and neuro-oncological clinics

**6. Entrustment for level of supervision necessary at each stage of training**

The entrustment of the professional activity should be adapted to local structures and educational frameworks.

**EPA 4: Postoperative management of brain tumour patients**

**1. Title of the EPA**

*Postoperative management of brain tumour patients*

**2. Specification and limitations**

The aim is to generate a systematic pathway for postoperative patients. This EPA includes standard post-operative management, detection, diagnosis and treatment of complications and later discharge and drawing up a patient – oriented treatment plan. Standard postoperative monitoring and evaluation should be initiated and performed independently. Postoperative complications and needs should be identified, diagnosed and treated promptly using and applying knowledge about basic principles of neuro-intensive care (ICP monitoring, ventilation etc.). Another focus of this EPA is the initiation of necessary postoperative investigations, drawing up an (interdisciplinary) therapy plan, and effective and appropriate communication of findings, prognoses and therapeutic options and risks with patients and their relatives. The proposed therapy plan should comply with the latest guidelines and take into account the patients’ wishes, safety and welfare.

**3. Most relevant domains of competence**

The concept of EPAs is a framework that aims to teach very specific competencies and professional activities rather than knowledge alone. Predefined professional activities should be taught and undertaken under direct supervision only until a standardized level of proficiency has been reached and both teacher and trainee are confident with the performance.

This EPA represents a key task of neuro-oncological practice. Competencies defined in table 1 apply to this EPA.

**4. Required knowledge, skills and attitude**

*Knowledge:*

- Extensive knowledge about the neuropathology of brain tumours including the impact of relevant molecular markers
- Extensive knowledge about signs, symptoms and management of complications/postoperative problems
- Diagnostic work-up and interdisciplinary treatment of patients with the first diagnosis of a brain tumour
- Basic knowledge about multimodal treatment plans, practical implementation and limitations of the different techniques
- Knowledge of the possibilities and limitations of radiation oncology treatment methods including stereotactic radiosurgery, proton therapy and intraoperative RT
- Consideration of common differential diagnoses of postoperative neurological deterioration and their individual prognosis
- Postoperative imaging of brain tumour patients (e.g. extent of tumour resection and detection of postoperative haemorrhage)

*Skills:*

- Systematic approach to patient assessment and therapy;
- Management of critically ill patients if applicable, (see EPA ‘Management of a deteriorating patient’)
- Excellent evaluation of post-operative imaging in terms of surgical outcome and possible complications, evaluations of possible residuals and calculation of volumes
- Initiation and coordination of interdisciplinary and -professional assessments, treatment and follow-up plans
- Adequate communication skills to initiate referrals
- Consultation and further (interdisciplinary) therapy
- Adequate collaboration in a multidisciplinary and -professional team
- Delivering bad news appropriately (resource-activating, supportive)
- Advising patients and their families on neurological and neurooncological diseases, their symptom load, prognosis, and operative and non-operative therapies based on up-to-date studies
- Considering the patient´s wishes

*Attitude:*

- Adequate and appropriate communication
- Constructive cooperation in a (multi-professional) team
- Recognition of own limitations and when to seek help from other team members to guarantee an optimal patient care
- Prioritization of urgent neuro-oncological / medical issues.

**5. Recommended potential assessment tools to evaluate progress and proficiency as a standardised, summative entrustment decision**

Tools used for assessment and documentation of progress as well as for a standardised, summative entrustment decision should be adapted to local structures and educational frameworks. However, according Bigg´s model (1996) of constructive alignment, assessments methods should align with the desired outcome (teaching of practical activities) and the teaching format used (EPAs). In our view, the following assessment tools might be suitable for this EPA:

- Direct observation of procedural skills (DOPS)
- Standardised feedback regarding management decisions from a neuro-oncological consultant and other medical professionals (e.g. consultant of other medical specialties, nurses)
- Supervisor feedback
- 360° feedback
- Patient feedback
- Chart review
- Procedure logbook
- Presentation and discussion of patients and their management in person, or over the phone
- Anticipatory guidance: “what if” discussion with a consultant to explore ability to cope with other scenarios.
- Knowledge may be tested by passing the U.S written boards or equivalent exam
- Learning outcome-based rotations in neuro-ICU, anaesthesia, neuro-pathology, clinical neurophysiology and neuro-oncological clinics

**6. Entrustment for level of supervision necessary at each stage of training**

The entrustment of the professional activity should be adapted to local structures and educational frameworks.

**EPA 5: Management of a deteriorating brain tumour patient**

**1. Title of the EPA**

Management of a deteriorating brain tumour patient

**2. Specification and limitations**

The aim is to use a systematic approach to manage deteriorating neuro-oncological patients, either in the emergency room (accidents & emergencies), in the intensive care unit, or on the hospital ward. A clear idea of appropriate differential diagnoses has to be formed and communicated, appropriate monitoring and investigations initiated if necessary, and treatment commenced. Frequent re-assessments of the patient should be undertaken to confirm diagnosis or re-consider its differentials.

**3. Most relevant domains of competence**

The concept of EPAs is used as a framework that aims to teach very specific competencies and professional activities rather than knowledge alone. Predefined professional activities should be taught and undertaken under direct supervision only until a standardised level of proficiency has been reached and both teacher and trainee are confident with the performance.

This EPA represents a key task of neuro-oncological practice. Competencies defined in table 1 apply to this EPA.

**4. Required knowledge, skills and attitude**

*Knowledge:*

- Medical, neuro-oncological and neurosurgical knowledge to assess the patient
- Recognise red flags and emergencies
- Consider common differential diagnoses
- Initiate time-critical further monitoring, assessment and therapy

*Skills:*

- Systematic approach to patient assessment, monitoring and therapy
- Skills in emergency medicine and neuro-oncological surgery managing deteriorating patients (e.g. the management of status epilepticus, cerebral oedema and ischemic stroke cerebral, herniation from a brain tumour with or without haemorrhage)
- Adequate judgement on the need of an implementation of further (invasive) monitoring / therapy
- Clear, concise and structured communication of the patient’s condition to a consultant, supervisor, or team member in a timely manner
- Adequate communication skills to initiate further assessments, referrals and the necessary (interdisciplinary) therapy; adequate collaboration in a multidisciplinary and -professional team.

*Attitude:*

- Recognition of a patient’s deterioration and usage of an appropriate level of urgency for further management according to its severity
- Recognition of own limitations and when to seek help from other team members to guarantee optimal patient care
- Prioritization of urgent neuro-oncological / medical issues
- Calm demeanour

**5. Recommended potential assessment tools to evaluate progress and proficiency as a standardised, summative entrustment decision**

Tools used for assessment and documentation of progress as well as for a standardised, summative entrustment decision should be adapted to local structures and educational frameworks. However, according to Bigg´s model (1996) of constructive alignment, assessments methods should align with the desired outcome (teaching of practical activities) and the teaching format used (EPAs). In our view, the following assessment tools might be suitable for this EPA:

- Direct observation of procedural skills (DOPS)
- Standardised feedback regarding management decisions from a neuro-oncological consultant and other medical professionals (e.g. consultant from other medical disciplines, nurses).
- Supervisor feedback
- 360° feedback
- Peer assessment
- Chart review
- Procedure logbook
- Presentation and discussion of patients and their management in person, or over the phone.
- Anticipatory guidance: “what if” discussion with a consultant to explore ability to cope with other scenarios
- Attending appropriate courses (e.g., of the German Society of Neurosurgery)
- Knowledge may be tested by passing the U.S written boards or equivalent exam
- Learning outcome-based rotations in neuro-ICU, anaesthesia, neuro-pathology, clinical neurophysiology and neuro-oncological clinics

**6. Entrustment for level of supervision necessary at each stage of training**

The entrustment should be adapted to local structures and educational frameworks. This EPA might be used as a tool to assess a resident´s ability to manage critically ill patients and subsequently a resident´s capability to be first on call.

**EPA 6: Early palliative care for dying patients and their families**

**1. Title of the EPA**

*Early palliative care for dying patients and their families*

**2. Specification and limitations**

The aim is to apply a systematic pathway for palliative and/or dying patients and their families. (Surgical) neuro-oncologists should feel responsible for palliative and dying patients and should specifically address their general needs and complex suffering. Furthermore, they should be able to discern a high symptom load in the physical, psychological, social or spiritual field which should in turn trigger a referral to the specialized palliative care.

**3. Most relevant domains of competence**

The concept of EPAs is a framework that aims to teach very specific competencies and professional activities rather than knowledge alone. Predefined professional activities should be taught and undertaken under direct supervision only until a standardized level of proficiency has been reached and both teacher and trainee are confident with the performance.

This EPA represents a key task of neuro-oncological practice. Competencies defined in table 1 apply to this EPA.

**4. Required knowledge, skills and attitude**

*Knowledge:*

- Basic understanding of the assessment and (pharmacological) treatment of symptoms and suffering on all four symptom levels (physical, psychological, social and spiritual) specific to the palliative and/or dying patient
- Basic understanding of the pharmacological treatment of symptoms and suffering specific to the palliative/dying patient
- Basic knowledge about palliative anti-tumour therapies (e.g. in the context of early integration or pain) and palliative care concepts (e.g. total-pain-concept)
- Recognize physical signs and symptoms of dying patients and conduct adequate management
- Criteria for when to start (specialized) palliative care (e.g. early integration)
- Basic knowledge about palliative care structures (e.g. specialized palliative care ward, specialized palliative care consultation, specialized outpatient palliative care, hospice care)

Skills:

- Advising patients and their relatives*;*
- Delivering bad news appropriately, using structured conversation models (e.g. SPIKES)
- Using resource-activating and supportive interventions to a reasonable extent in seriously ill patients (e.g., holding and containing)
- Taking an active and considerate role in a multidisciplinary and multi-professional medical team with high quality and effectiveness;
- Performing a standardized symptom assessment (e.g. MIDOS)
- Coordinating interdisciplinary and -professional assessments and drawing up treatment plans with special regard to the four symptom levels (physical, psychological, social and spiritual)
- Recognizing dying patients and treating them within standardized procedures (e.g. withdrawal of unnecessary medication, prescription of essential medication for symptom control of frequent symptoms in the dying phase and information of close relative and the patient’s GP)

Attitude:

- Adequate and appropriate communication
- Cooperation in a (multi-professional) team and constructive teamwork
- Appreciating the importance and time sensitivity in treating palliative and dying humans;
- Establish ethical principles and apply them to end-of-life care

**5. Recommended potential assessment tools to evaluate progress and proficiency as a standardised, summative entrustment decision**

Tools used for assessment and documentation of progress as well as for a standardised, summative entrustment decision should be adapted to local structures and educational frameworks. However, according Bigg´s model (1996) of constructive alignment, assessments methods should align with the desired outcome (teaching of practical activities) and the teaching format used (EPAs). In our view, the following assessment tools might be suitable for this EPA:

- Direct observation of procedural skills (DOPS)
- Supervisor feedback
- 360° feedback
- Patient/relative feedback
- Chart review
- Procedure logbook
- Anticipatory guidance: “what if”’ discussion with a consultant to explore ability to cope with other scenarios.
- Knowledge may be tested by passing the U.S written boards or equivalent exam

**6. Entrustment for level of supervision necessary at each stage of training**

The entrustment of the professional activity should be adapted to local structures and educational frameworks.

**EPA 7: Collaboration as a member of an interdisciplinary and / or -professional neuro-oncology** **team**

**1. Title of the EPA**

*Collaboration as a member of an interdisciplinary and / or -professional neuro-oncology team*

**2. Specification and limitations**

The aim of this EPA is to qualify trainees as a professional member of an interdisciplinary and/or -professional team. Professionals in neuro-oncology should actively strive to integrate into such teams and should prioritize optimal patient care and team needs over personal needs.

**3. Most relevant domains of competence**

The concept of EPAs is a framework that aims to teach very specific competencies and professional activities rather than knowledge alone. Predefined professional activities should be taught and undertaken under direct supervision only until a standardized level of proficiency has been reached and both teacher and trainee are confident with the performance.

Neuro-oncology is a highly interdisciplinary sub-speciality and is characterized by a high degree of interprofessional work. Therefore, collaboration as a member of a multidisciplinary team represents a key element of neuro-oncological practice. The competencies defined in table 1 are most applicable for this EPA.

**4. Required knowledge, skills and attitude**

*Knowledge:*

- Factors that affect teamwork and effective communication, strategies for safe communication (eg.pilot-training)

*Skills:*

- Actively strives to integrate into the team,
- Adequate and appropriate communication, even in emotionally challenging situations
- Establish a climate of respect, appreciation, integrity, and trust

*Attitude:*

- Feels committed to the goal of the team and optimal patient care;
- Prioritizes an optimal patient care and team needs over personal needs;
- Recognizes the role, responsibilities, contributions and value of all team members,
- Knows own limitations and if feasible requests help from other team members to guarantee an optimal patient care
- includes and attentively listens to all team members and considers feedback and ideas
- Offers help to members of the team in need
- Shares knowledge and know-how with team members clearly and understandably
- Is actively involved in enrolling patients into clinical trials

**5. Recommended potential assessment tools to evaluate progress and proficiency as a standardised, summative entrustment decision**

Tools used for assessment and documentation of progress as well as for a standardised, summative entrustment decision should be adapted to local structures and educational frameworks. However, according Bigg´s model (1996) of constructive alignment, assessments methods should align with the desired outcome (teaching of practical activities) and the teaching format used (EPAs). In our view, the following assessment tools might be suitable for this EPA:

- Standardised feedback regarding collaboration in multidisciplinary teams
- Direct observation of procedural skills (DOPS)
- Courses on team resource management
- Attending/presenting at tumour board meetings to be part of multi-disciplinary discussions.
- Learning outcome-based rotations in neuro-ICU, anaesthesia, neuro-pathology, clinical neurophysiology and neuro-oncological clinics

**6. Entrustment for level of supervision necessary at each stage of training**

Depending on local structures and educational concept: The resident’s entrustment level might be assessed by neuro-surgical consultants, using the above information. This EPA represents an essential part of surgical neuro-oncological work and is therefore crucial in neuro-oncological training.

**EPA 8: Basic and Clinical Research activity in Neuro-Oncology**

**1. Title of the EPA**

*Basic and Clinical Research activity in Neuro-Oncology*

**2. Specification and limitations**

The aim is to provide trainees with a systematic/standardized approach to both basic and clinical research applied to Neuro-Oncology. Every specialist will be able to master all the key concepts and the leading research concepts that define Neuro-Oncology and represent the challenges to be faced by the next generation in this speciality.

**3. Most relevant domains of competence**

The concept of EPAs is a framework that aims to teach very specific competencies and professional activities rather than knowledge alone. Predefined professional activities should be taught and undertaken under direct supervision only until a standardized level of proficiency has been reached and both teacher and trainee are confident with the performance.

This EPA represents a key task of neuro-oncological practice. Competencies defined in table 1 apply to this EPA.

**4. Required knowledge, skills and attitude**

*Knowledge:*

- Basic understanding of the main scientific challenges in Neuro-Oncology
- Basic knowledge about basic and clinical research methods and approaches applied to Neuro-Oncology
- Basic knowledge about data management, statistical analysis and interpretation of data

Skills:

- Perform a basic and advanced literature review based on common scientific libraries (e.g. PubMed)
- Translate problems into precise scientific questions and generate scientific hypotheses based on it
- Compile, analyse und interpret clinical and experimental data sets, appropriate use of statistical methods for testing hypotheses
- Conduct and interpret basic/intermediate laboratory experiments in Neuro-Oncology
- Setting up a clinical trial (as investigator or principal investigator) or joining it as a collaborating partner
- Conduction of clinical trials according to established ethical standards, protocols, national laws and international guidelines (e.g. Good clinical practice)
- Prepare scientific results for a specialist audience according to the rules of scientific publications and present them to a specialist audience or lay people, being able to present at scientific meetings

Attitude:

- Adequate and appropriate communication
- Cooperation in a (multi-professional) team and constructive teamwork
- Appreciating the importance of both basic and clinical research; assess, apply and translate new knowledge and practices
- Establish ethical principles and apply them in research in Neuro-Oncology
- Knowledge, respect of and adherence to established ethical protocols, national laws and international guidelines (e.g. declaration of Helsinki), adherence to guidelines of good scientific practice and good clinical practice
- Harvesting tumour tissue for biobanking and enrolment into clinical trials
- Respect of and adherence to national and international law and guideline that regulate animal testing, prefer use of non-animal methods if possible (replacement), limit number of experimental animals (reduction) and minimize potential suffering, pain and distress, enhance experimental animal welfare for the animals used (refinement)
- Up-to-date knowledge of latest research and landmark papers

**5. Recommended potential assessment tools to evaluate progress and proficiency as a standardised, summative entrustment decision**

Tools used for assessment and documentation of progress as well as for a standardised, summative entrustment decision should be adapted to local structures and educational frameworks. However, according Bigg´s model (1996) of constructive alignment, assessments methods should align with the desired outcome (teaching of practical activities) and the teaching format used (EPAs). In our view, the following assessment tools might be suitable for this EPA:

- Direct observation of procedural skills (DOPS)
- Supervisor feedback
- 360° feedback
- Regular attendance of (international) Meetings attendance and presentation of own results
- Publication track-record.

**6. Entrustment for level of supervision necessary at each stage of training**

The entrustment of the professional activity should be adapted to local structures and educational frameworks.
